# Advancing theory development: exploring the leadership–climate relationship as a mechanism of the implementation of cultural competence

**DOI:** 10.1186/s13012-017-0666-9

**Published:** 2017-11-14

**Authors:** Erick G. Guerrero, Karissa Fenwick, Yinfei Kong

**Affiliations:** 1 Suzanne Dworak-Peck School of Social Work, University of Southern California, 655 West 34th Street, Los Angeles, CA 90089 USA; 2 Marshall School of Business, University of Southern California, 655 West 34th Street, Los Angeles, CA 90089 USA; 3 Mihaylo College of Business and Economics, California State University, Fullerton, Fullerton, CA 90089 USA

**Keywords:** Transformational leadership, Organizational climate, Cultural competence, Implementation

## Abstract

**Background:**

Leadership style and specific organizational climates have emerged as critical mechanisms to implement targeted practices in organizations. Drawing from relevant theories, we propose that climate for implementation of cultural competence reflects how transformational leadership may enhance the organizational implementation of culturally responsive practices in health care organizations.

**Methods:**

Using multilevel data from 427 employees embedded in 112 addiction treatment programs collected in 2013, confirmatory factor analysis showed adequate fit statistics for our measure of climate for implementation of cultural competence (Cronbach’s alpha = .88) and three outcomes: knowledge (Cronbach’s alpha = .88), services (Cronbach’s alpha = .86), and personnel (Cronbach’s alpha = .86) practices.

**Results:**

Results from multilevel path analyses indicate a positive relationship between employee perceptions of transformational leadership and climate for implementation of cultural competence (standardized indirect effect = .057, bootstrap *p* < .001). We also found a positive indirect effect between transformational leadership and each of the culturally competent practices: knowledge (standardized indirect effect = .006, bootstrap *p* = .004), services (standardized indirect effect = .019, bootstrap *p* < .001), and personnel (standardized indirect effect = .014, bootstrap *p* = .005).

**Conclusions:**

Findings contribute to implementation science. They build on leadership theory and offer evidence of the mediating role of climate in the implementation of cultural competence in addiction health service organizations.

## Background

Health care organizations require leadership to implement practices that are effective and culturally responsive to the increasing racial and ethnic diversity of the US population. To address the well-established disparities between health outcomes of racial and ethnic minorities compared to Whites, federal and private institutions have supported the implementation of culturally competent practices [1, 2]. Cultural competence refers to the recognition and responsiveness of organizations to the service needs of culturally and linguistically diverse populations and aims to improve health care quality, engage racial and ethnic minority clients in care, and reduce outcome disparities [3–6]. Some culturally responsive practices, such as language and racial and ethnic provider–client matching, translating materials, and using cultural stories to engage clients in services, have robust associations with health outcomes [3–6]. Yet culturally responsive practices, which are consistent with Klein and Sorra’s [7] theory of innovation implementation, can be considered an innovation because their implementation require active and coordinated use by many organizational members and they are not routinely applied in health care settings. Efforts to systematically examine the drivers of implementation of innovative culturally responsive practices in health care [8] and addiction health services [9, 10] are limited.

Leadership is a key factor associated with implementation of service innovations such as cultural competence, given that organizational leaders are generally responsible for overseeing the implementation process [11]. Theory suggests that leadership affects implementation both directly and indirectly by shaping the organizational context, which then influences employee behaviors [12]. Developing research shows that leaders’ communication and prioritization of new norms and expectations (e.g., safety) influence employee adoption of those norms and endorsement of congruent practices (e.g., safer work practices), generally referred to as organizational climate. The organizational climate supports and encourages employees in implementing a new practice [13]. This leader–climate–practice mechanism has been examined in the context of implementation of industrial safety [14], corporate customer services [15], and evidence-based health care practices [16]. However, it is critical to examine the extent to which this mechanism applies to cultural competence. Although commonly endorsed in health care services, cultural competence requires creative thinking to implement key cultural aspects (e.g., familismo, language, context) that may affect client outcomes [1, 2]. Understanding the role of leadership and implementation climate in the uptake of cultural competence in health care is critical to improve clients’ response to treatment, particularly in substance use disorder treatment programs whose unique structure as generally small programs with an average of five to six employees allows managing leaders to have direct and daily interactions with employees.

### Theoretical framework

One of the most influential leadership styles described in the management literature is transformational leadership [17, 18]. Transformational leadership is a leader’s ability to inspire others to follow a particular course of action and perform beyond previous levels [19, 20]. Transformational leaders consider the unique talents of each staff member or employee (these terms are used interchangeably in this paper), give specific feedback to each staff member based on his or her needs, stimulate new ways of solving problems, and create a shared sense of purpose among all staff members [19, 21]. Transformational leadership is thought to be transmitted through a leader’s expression of his or her values and goals and selection, modeling, and communication of relevant information, which staff members use when weighing options and making decisions [16, 22, 23].

Transformational leadership has been shown to play a role in the adoption and implementation of innovations in health care [24–26]. Leadership style influences multiple organizational processes involved in delivery of innovative practices [11]. For instance, leaders initiate adoption decisions, develop strategic goals and activities supporting innovation implementation, secure necessary resources, build organizational capacity for change, scan the internal and external contexts, and provide performance feedback to the staff [9, 27, 28]. One of the most important ways in which leaders affect delivery of innovative practices is by creating an organizational context conducive to implementing new practices. In particular, leaders positively or negatively contribute to the creation, development, and sustainment of an organizational climate that fosters employee attitudes and behaviors that support innovative practice use [25, 29, 30]. A leader in the current study refers to executive or upper level directors who oversee the overall operation of treatment programs and whose transformational style may influence employees, such as middle managers (i.e., supervisors) and counselors.

#### Organizational climate

Organizational climate can be understood as employees’ shared perceptions of procedures, practices, and behaviors that are rewarded and supported by management with a specific purpose [31]. When the purpose involves implementing practices that respond to client’s native cultural norms and values, language, and history, the resultant shared perceptions can be defined as climate for implementation of cultural competence.

Many definitions of climates have emerged. These definitions of organizational climates seem to differ based on the language used to capture the level of agreement among employees regarding the implicit and or explicit priorities of the organization. These priorities are generally communicated by managers through organizational policies, procedures, and practices, leading to employees’ shared notion of “the way things are done around here” [32, 33]. Hence, measurement of this conceptualization has focused on what is expected, supported, promoted, rewarded, and punished in the work context [34, 35].

Moreover, organizational climate can be conceptualized and measure either as a singular, molar climate (e.g., shared perceptions of organizational policies and procedures) that influences nearly all activities in an organization or as multiple simultaneous focused climates (shared perceptions of a direct manager’s priorities through rewards and support of employees’ specific behaviors and attitudes) [35]. Recent climate research suggested that a molar climate may lay the groundwork for focused climates, which then serve as more proximal predictors of outcomes [36, 37]. As a result, the latest research on climate has focused on the development of specific climates for desired organizational goals (such as customer service and safety) or processes (such as creativity) [15]. Focused climates consist of the components of the organizational environment that are most influential in orienting employee behavior toward the outcome of interest. The development of these focused climates improves prediction and understanding of targeted outcomes and makes the climate construct more practically relevant to managers seeking to enhance performance in specific areas [15, 37]. Assessment of focused climates using employees’ shared perceptions of the consistency among policies, procedures, and practices—basically “leader’s words and actions” in prioritizing implementation of a new practice—may improve understanding of climate as a mechanism that influences employees’ attitudes and behaviors [35].

Empirical research has supported the association between many specific climates and their targeted outcomes. Safety climate is associated with decreased accidents [38, 39], service climate is associated with greater customer satisfaction [40, 41], climate for creativity is associated with creative performance [42], and climate for innovation is associated with greater innovative behavior [43]. In this study, implementation climate for cultural competence can be defined as employees’ shared perceptions of their program supervisors’ priority to implement culturally responsive practices through expectations, support, promotions, rewards, and punishments [34, 35]. In a strong implementation climate, employees perceive new practices as a priority rather than a distraction or disruption [7, 44]. Several studies have found a positive association between implementation climate and implementation effectiveness, although empirical studies of implementation climate are limited [45–47].

Implementation climate can be further tailored to refer to implementation of new knowledge, practices, or processes as promoted, rewarded, and expected by direct supervisors and perceived by employees. For example, in a strong climate for implementation of cultural competence, employees perceive that the adoption, implementation, and use of culturally competent knowledge, services, and practices is expected, rewarded, and supported by management [48].

#### Organizational climate as a mediator

Leading climate scholars [35, 37, 49] have advised researchers to examine the relationships among leadership, climate, and outcomes in more depth. They recommended exploring how leaders create and maintain climate and how climate mediates the relationship between leadership and outcomes.

Understanding the leader–climate–practice mechanism necessitates a theoretical explanation of how leaders influence focused climates. Leaders may shape organizational climate through a social learning process in which staff members repeatedly interact with and observe their leader to interpret organizational priorities [29, 50]. Leaders convey the importance of various tasks in an organization through implicit and explicit communication of priorities [51, 52]. Through their behavior and interactions with employees, they communicate the value of each task and their evaluation of tasks in comparison with one another. Leaders communicate their priorities in several ways. They develop strategic goals for the organization, disseminate information, monitor and supervise staff activities, model desired behavior, and reward staff behavior in line with the prioritized behavior or outcome [53–55]. These activities occur more frequently or intensely for prioritized behaviors or outcomes in comparison to those that are not prioritized.

**Fig. 1:**
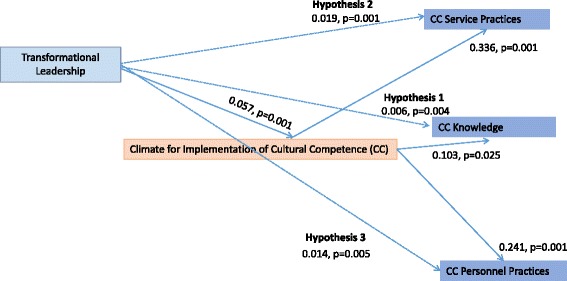
Path analysis of transformational leadership, climate for implementation of cultural competence, and three culturally competent practices. *Note.* Control variables include program funding, licensure, and professionalism

Three main attributes characterize the relationship between leadership behavior patterns and communicated priorities [56]. Pattern orientation refers to communicating a particular priority relative to other competing interests, pattern variability refers to the consistency of leader behavior in communicating a particular priority over time and among different staff members, and pattern simplicity is the number of contingencies that influence a priority. Leaders who prioritize implementation of cultural competence may communicate this priority by developing strategic goals and plans supporting culturally competent practices, allocating resources for culturally competent services, and providing supervision and coaching to build culturally competent knowledge. In addition, they may persevere in the face of challenges to implementation and reward employees based on provision of culturally competent services [27, 28, 57]. By communicating their expectations and priorities in these ways, leaders develop, support, and perpetuate an organizational climate [56]. See Fig. 1.

The climate–practice relationship is based on climate’s role as a guide and sense-making mechanism that influences employees’ attitudes and behaviors [58]. Employees interpret their environment, and climate acts as a critical determinant that influences these interpretations [59, 60].

### Overview of the current research

We examined the organizational processes driving implementation of culturally competent knowledge, practices, and services. We focused on the organizational climate for implementation of cultural competence (employees’ shared perceptions of middle managers’ priorities, expectations and rewards to implement cultural competence) to explore the extent to which it enhances executive leaders’ transformational leadership in the implementation of significant culturally competent practices in addiction health services. Executive or program directors are referred to here as executive or upper level leaders, whereas clinical supervisors are referred to as middle managers. Our hypotheses are as follows:

Hypothesis 1

Transformational leadership will be positively and indirectly related to the implementation of culturally competent knowledge through climate for implementation of cultural competence.

Hypothesis 2

Transformational leadership will be positively and indirectly related to the implementation of culturally competent service practices through climate for implementation of cultural competence.

Hypothesis 3

Transformational leadership will be positively and indirectly related to the implementation of culturally competent personnel practices through climate for implementation of cultural competence.

## Methods

### Background and context

This study used data from a larger study featuring a sampling frame of 408 addiction health services programs funded by a public health department in Los Angeles County between July 1, 2013, and December 31, 2013. Data for the current study came from the second wave of the larger study, during which all relevant variables were introduced. The initial sampling procedure involved a random selection of 147 outpatient programs located in communities with a population composition of 40% or more Latino or African American residents or both in the study region. Data collection for this wave included information from an average of three direct-service providers per program (one manager and two counselors). Only programs with at least two respondents, a counselor, and a manager (either director or supervisor) were included in the analysis. Most programs had an executive or upper level director, a middle manager (e.g., supervisor), and a counselor. Only 5% of programs had one manager with dual directorial and supervisory responsibilities and who reported on both program funding and regulation, as well as on the transformational leadership of their executive director. Data collectors obtained staff rosters to select at random respondents within programs; overall, the sample composition was 25.4% directors, 15.7% supervisors, and 58.9% counselors.

Considering eligible and still operating programs, the final analytic sample featured 112 programs (92% response rate) and 427 individual participants. The 35 programs not included in this wave did not differ from the original analytic sample in terms of main independent variables, such as leadership style (transformational) and cultural competence (*p* > .05).

A treatment program was defined as an outpatient site managed as a standalone program or by a parent organization generally situated at a different location. The average age of participants in the sample was 46 years, and 34% of participants were men. Most managers were African American (45%) or Latino (32%), as were counselors (43 and 47%, respectively).

Our power analysis suggest that we would have 80% power to detect a medium effect size, which is associated with a Pearson’s correlation of .24, when considering 99 programs and 15 variables in the statistical regression analysis [61]. To increase the validity of the survey measures, we conducted in vivo observations and reviewed printed materials available at each provider site (e.g., brochures, group activities, posted signs). For example, for each dependent variable (culturally competent practices), we used a matrix (Excel sheet) with key program features to cross-check the consistency of staff reports on survey measures. All measures were responded to by supervisors and counselors except for regulation, public funding, and professionalization, which were responded to by upper level managers (i.e., directors) or managers with both supervisory and directorial responsibilities.

### Dependent variables

The organizational cultural competence survey measures were adapted from the Cultural Competence Self-Assessment Questionnaire [6]. This 57-item measure is composed of six subscales assessing culturally competent practices: (a) knowledge of, (b) outreach to, and (c) personal involvement in racial and ethnic minority communities; (d) development of resources and linkages to serve racial and ethnic minorities; (e) development of policies and procedures to effectively respond to the service needs of racial and ethnic minority patients; and (f) hiring and retention of employees with racial and ethnic minority backgrounds (for a full description of items, see Mason [6]). We used the 57 items to empirically develop three critical subscales as suggested by the literature: knowledge (eight items, e.g., Do you know the prevailing beliefs, customs, norms, and values of Latinos in your service area?); service practices (nine items, e.g., Does your agency utilize interpreters to work with limited English-proficient Latinos?); and personnel practices (nine items, e.g., Does your agency utilize interpreters to work with limited English-proficient Latinos). Responses were rated on a 4-point Likert scale (1 = not at all to 4 = often) and averaged to create total scores for each subscale. Higher scores indicated higher levels of cultural competence in each subdomain, as perceived by supervisors and counselors. Confirmatory factor analysis was conducted to validate these measures (see Table 1 for descriptive statistics; confirmatory factor analysis results are reported in Table 2).

**Table 1:** Descriptive statistics from 427 participants nested in 112 programs

	*M* (SD) or %	Range
Participant demographics		
Age	46.6 (11.7)	19–75
Race and ethnicity		
Latino	40.1	
African American	27.6	
White	21.6	
Other	10.1	
Position		
Director	25.3	
Supervisor	15.7	
Counselor	58.8	
Education		
High school or lower	5.9	
College	56.4	
Graduate degree	32.3	
Program measures		
Transformational leadership	39.4 (7.5)	10–50
Climate for implementation of cultural competence	3.4 (0.85)	1–5
Knowledge	2.9 (0.57)	1–4
Personnel practices	2.6 (0.74)	1–4
Service practices	2.4 (0.87)	1–4
Control variables		
Percentage of public funding	67.9	
Licensing	96.3	
Professional accreditation (Joint Commission)	32.7	
Professionalization (% of staff with graduate degree)	21.6 (18.3)	0–75

**Table 2:** Confirmatory factor analysis of culturally competent practices (knowledge, service, and personnel)

	Obs	Item test	Item rest	Interitem covariance	*α*
Knowledge					
How well are you able to describe differences within various Latino or Hispanic groups?	424	.701	.557	.292	.888
How well are you able to describe the strengths of Latino groups in your service area?	427	.807	.716	.277	.861
How well are you able to describe the social problems of Latino groups in your community?	425	.866	.804	.270	.849
Do you know the prevailing beliefs, customs, norms, and values of Latinos in your service area?	426	.834	.749	.267	.856
Do you know the social service needs of Latinos that go unaddressed by the formal social service system?	421	.829	.736	.261	.857
Do you know how the causes of mental illnesses are viewed by Latino groups in your area?	430	.773	.656	.274	.870
Full scale				.274	.884
Service practices					
Does your agency use Latino-specific assessment instruments for diagnosis?	294	.737	.647	.613	.832
Does your agency use Latino culture-specific treatment approaches?	290	.761	.683	.605	.830
Does your agency envision community empowerment as a treatment goal?	291	.745	.660	.602	.831
Does your agency review case practice on a regular basis to determine relevancy to clients of color?	288	.749	.659	.594	.831
Does your agency provide or facilitate child care?	298	.688	.569	.612	.842
Does your agency provide or facilitate transportation (e.g., bus tickets, ride sharing)?	296	.627	.495	.632	.850
Does your agency include clients’ families and community in services?	298	.734	.637	.599	.833
Does your agency translate agency materials into Spanish?	302	.634	.520	.640	.846
Does your agency offer payment arrangements for indigent or low-income clients?	296	.499	.361	.688	.860
Full scale				.621	.855
Personnel practices					
Does staff utilize cultural consultants who can help them work more effectively?	422	.516	.375	.486	.863
Does your agency provide training that help staff work with Latinos?	419	.570	.445	.475	.856
Does your agency utilize interpreters to work with limited English-proficient Latinos?	418	.503	.346	.485	.869
In your program, are there Latinos represented in managerial and administrative positions?	424	.759	.670	.421	.834
In your program, are there Latinos represented in direct-service positions?	426	.709	.620	.444	.840
In your program, are there Latinos represented in administrative support positions?	421	.770	.691	.425	.832
In your program, are there Latinos represented in board positions?	413	.789	.712	.418	.830
In your program, are there Latinos represented in agency consultants?	410	.816	.750	.416	.827
In your program, are there Latinos represented in subcontractors?	400	.711	.711	.420	.831
Full scale				.443	*.858*

Obs observations

### Independent variables

Our main independent variables were leadership style (transformational) and climate for the implementation of cultural competence. A 7-item measure assessed transformational leadership among executive or upper level leaders (i.e., agency directors) [62]. This short version has been used in several studies on staff’s perception of directors’ transformational leadership in health care services [62–65]. Each director’s leadership was rated by clinical supervisors and counselors on a 5-point scale (1 = strongly disagree to 5 = strongly agree) and scores were totaled as suggested by the measure’s authors [62]. Higher scores represented higher transformational leadership capacity as perceived by clinical supervisors and counselors. Cronbach’s alpha for transformational leadership capacity was .92.

We measured climate for the implementation of cultural competence using six items. The development of these items was informed by other climate measures in safety [38], customer satisfaction [41], and innovative behavior [43]. Climate is generally enforced through middle managers’ communicated priorities, rewards, and expectations [36, 56]. Altogether, the leadership–climate–practice mechanism is supported by empirical evidence showing that organizational climate acts as a mediator between leadership and a variety of outcomes, including innovation implementation behavior [25, 30, 59, 60]. Our measure of climate was rated by clinical supervisors and staff members on a 5-point scale (1 = not at all to 5 = very well) and scores were aggregated by program and totaled as suggested by authors of climate measures [35, 38, 49]. Higher scores represented stronger employee perceptions that the implementation of culturally competent practices was promoted, rewarded, and expected by their manager. A sample item of a promoted practice is: “Supervisor prioritizes enhancing the staff’s cultural competence by helping resolve cross-cultural issues with clients.” An item measuring a rewarded practice include: “Supervisor provides incentives to the staff to become linguistically and culturally responsive, despite investment of time and resources.” An item measuring an expected practice is: “Supervisor has emphasized the importance of translation of material and development of policies and procedures to respond to clients with limited English proficiency, despite her or his other multiple responsibilities.” We measured the internal consistency of survey items to determine their overall reliability as a measure. Cronbach’s alpha for this leadership measure was .88.

### Control variables

These variables included regulation, public funding, and professionalization. Please refer to Table 1 for a full list of variables. We assessed regulation by determining whether each program had a state license and accreditation by the Joint Commission. We also included a measure of percentage of public revenue in each program’s budget; professionalization was measured using the percentage of staff members with a graduate degree. These factors are associated with implementation of new practices in addiction health services [26, 63, 66].

### Statistical analyses

Studies have shown that leadership across organizations is nested in different settings [67], requiring adjustment for context to properly evaluate leadership influence. In this case, hierarchical linear modeling was needed to analyze nested data [68]. In our study, we accounted for control variables, program funding, license, professional accreditation, and professionalism, which play a role in the implementation and delivery of new practices [26, 69]. The hierarchical linear modeling analysis considered staff members at level 1 to be nested in programs at level 2.

As reported in other studies using these data [65], because we assumed data was missing at random, we relied on maximum likelihood estimation in multivariate regressions, which allows us to obtain unbiased estimate parameters [70]. Our highest rate of missing data for any given variable was 4%. This procedure was conducted in Stata/SE version 12.

Confirmatory factor analysis was conducted in Stata/SE using maximum likelihood estimation procedures as well to validate our measure of climate for the implementation of cultural competence consistent with other organizational level research [71]. The results of the analysis are shown in Tables 2 and 3.

**Table 3:** Psychometric properties of climate for the implementation of cultural competence

Item	Obs	*M* (SD)	ICC	Awg	*α*
Item 1: Supervisor provides incentives to the staff to become linguistically and culturally responsive, despite investment of time and resources	237	2.916 (1.121)		0.596 (0.434)	.885
Item 2: Supervisor creates opportunities to talk to the staff about ways to respond to clients’ cultural and linguistic service needs despite his or her other administrative responsibilities (e.g., documentation and billing)	239	3.410 (1.045)		0.665 (0.285)	.847
Item 3: Supervisor provides clear guidance about how to respond to clients’ cross-cultural issues despite busy schedule	241	3.577 (1.014)		0.602 (0.410)	.829
Item 4: Supervisor has emphasized the importance of translation of material and development of policies and procedures to respond to clients with limited English proficiency, despite her or his other multiple responsibilities	238	3.378 (1.043)		0.540 (0.449)	.845
Item 5: Supervisor prioritizes enhancing the staff’s cultural competence by helping resolve cross-cultural issues with clients	240	3.546 (0.967)		0.636 (0.453)	.850
Test scale			.07	0.774 (0.221)	.878

To validate our measure of climate for the implementation of cultural competence and determine whether it is a unit-level construct, we relied on established methodologies applied to organizational constructs following the approach used by Glisson and James ([72], p. 780). First, we used 'confirmatory factor analysis to determine whether responses justify a latent concept of *climate for implementation of cultural competence*. Second, we conducted a within-group consistency analysis using *r*_WG_ to test whether members of each of the *112 treatment programs* agreed with one another in their responses to the *leadership scales* [73]. This approach is common in organizational research to justify whether individual level responses can be aggregated and represent program measures. Third, we conducted between-group analysis using eta squared via analysis of variance and intraclass correlation coefficient via hierarchical linear modeling to test between-group differences among *program staff members* for each construct.' [74].

Finally, we used hierarchical linear modeling path analyses in Stata version 13. We selected a random-intercepts model to estimate relationships between individual-level measures nested in programs [68, 75]. Specifically, we ran three regression models, one per outcome, while controlling for program regulation, funding, and professionalism to estimate the contribution of transformational leadership to cultural competence outcomes via climate for the implementation of cultural competence. Our analyses used individual measures while controlling for staff measures embedded in programs. The path model was implemented using the GSEM builder in Stata/SE version 12. This model studied both the direct effects of the causal variable (i.e., transformational leadership) on the outcome (culturally responsive practices) and its indirect effects through the mediator (implementation climate). These analyses respond to the main research question: To what extent does climate for implementation of cultural competence play a mediating role in the relationship between transformational leadership and program implementation of cultural competence (knowledge, services, and personnel practices)?

## Results

We conducted confirmatory factor analysis to validate our outcome measures of cultural competence and mediator variable of climate for implementation of cultural competence. The 57-item measure of cultural competence [6] resulted in three dimensions of organizational cultural competence—knowledge (six items), service practices (nine items), and personnel practices (nine items)—that are consistent with theoretical and empirical descriptions of this concept [4–6]. See Table 2.

### Cultural competence: knowledge

We excluded two of the original eight items because they had item-rest correlations less than .50, resulting in a six-item scale. As shown in Table 2, Cronbach’s alpha for this scale was very high (.88), with all items contributing in the same direction and item-test and item-rest correlations greater than .56. Therefore, for ease of interpretation, we averaged the six items to create a new aggregate measure.

### Cultural competence: service practices

After inconsistent results, we reduced the original 14 items to nine that contributed in the same direction and had consistent levels of item-test and item-rest correlations, with only one less than .57. As seen in Table 2, Cronbach’s alpha for this scale was high (.86). We created a new aggregated scale to measure culturally competent service practices by averaging the final nine items.

### Cultural competence: personnel practices

We excluded seven of the original 16 items because they had inconsistent item-test (< .74) and item-rest (< .67) correlations. As shown in Table 2, the resulting nine-item scale had a high Cronbach’s alpha (.86) and all items contributed in the same direction. As in the previous measures, these items were averaged to create a new aggregate measure.

### Climate for implementation of cultural competence

As seen in Table 3, Cronbach’s alpha for midlevel leaders’ prioritization of implementing cultural competence was very high (.88). In addition, all items contributed in the same direction and had item-test and item-rest correlations greater than .57. Given this information and for ease of interpretation, we created a new measure by averaging our items representing promoted, rewarded, and expected culturally competence practices.

### Within- and between-group analyses

We computed indexes of within- and between-program consistency of responses for the measure of program climate for implementation of cultural competence. We calculated *r*_WG_ values to assess within-program consistency and found an average *r*_WG_ of .72 (not reported in tables), indicating a high level of consistency of responses within programs. We calculated intraclass correlation coefficients and reviewed the eta-squared values to determine between-program differences of .68. The intraclass correlation coefficient (type 1 indicates the proportion of total variance between programs, whereas eta squared indicates the proportion of total variation between programs [68, 72, 76, 77]. Type 1 intraclass correlation coefficient values (.07 in this study) are typically less than .20 and usually smaller than eta-squared values [76] supporting that program membership contributed to the resulting estimates. The consistency in within-program responses and discrepancy in between-program variance justified aggregating individual-level responses to the program level on measures of climate for implementation of cultural competence. This program-level measure was included in the following cross-level analyses of relationships between individual-level and program-level variables.

### Path analysis: hypothesis testing

Results from each of the three path regression analyses indicated that transformational leadership was indirectly associated with implementation of three culturally competent practice outcomes through climate for implementation of cultural competence. See Fig. 1. We describe the results of the path analysis based on the three study hypotheses.

We found support for hypothesis 1, which posited that transformational leadership would be positively and indirectly related to the implementation of culturally competent knowledge through climate for implementation of cultural competence. Employees’ and supervisors’ higher ratings of their director’s transformational leadership were indirectly associated with higher degree of implementation of culturally competent knowledge (standardized indirect effect = .006, bootstrap *p* = .004).

We found support for hypothesis 2, which posited that transformational leadership would be positively and indirectly related to the implementation of culturally competent service practices through climate for implementation of cultural competence. Employees’ and supervisors’ higher ratings of their director’s transformational leadership were indirectly associated with higher degree of implementation of service practices (standardized indirect effect = .019, bootstrap *p* < .001).

We found support for hypothesis 3, which posited that transformational leadership would be positively and indirectly related to the implementation of culturally competent personnel practices through the role of climate for implementation of cultural competence. Employees’ and supervisors’ higher ratings of their director’s transformational leadership were indirectly related to higher degree of implementation of culturally competent personnel practices (standardized indirect effect = .014, bootstrap *p* = .005). The main mediation models presented adequate fit statistics (*χ* ^2^ = 127.20, *df* = 22, *p* < .001, RMSEA < .001, CFI ≈ 1.000, TLI ≈ 1.000).

Compared with the indirect relationships found, the analysis highlighted stronger direct relationships among variables of interest. Transformational leadership was positively associated with climate for implementation of cultural competence (standardized indirect effect = .057, bootstrap *p* < .001). In particular, the relationship between climate for implementation of cultural competence and practices were robust—climate was positively associated with knowledge (standardized direct effect = .103, bootstrap *p* = .025), service practices (standardized direct effect = .336, bootstrap *p* < .001), and personnel practices (standardized direct effect = .241, bootstrap *p* < .001).

## Discussion

The current study examined three areas: (a) exploration of a focused climate and development of an associated measure, climate for implementation of cultural competence; (b) development of culturally competent practices measures; and (c) examination of the model (relationships between leadership and these new measures). Findings highlight the importance of focused climates in maximizing the influence of transformational leadership on employees’ implementation of congruent practices in health care. The measure representing climate for implementation of cultural competence had adequate psychometric properties and was further used to test a conceptual framework of the role of leadership in implementation processes in addiction health services organizations.

The culturally competent practices measures had adequate psychometric properties. The three measures (knowledge, services, and personnel practices) represented main areas of cultural competence in health care [3–5]. The examination of the relationships between leadership and these new program measures revealed associations among employee perceptions of executive or upper level directors leadership style, perceptions of middle managers’ expectations, promotion, and rewards regarding the implementation of cultural competence (climate), and their reported implementation of increased knowledge about racial and ethnic minority communities, culturally tailored service practices, and culturally tailored personnel practices. We found that directors’ transformational leadership influenced supervisors’ expectations to implement cultural competence and that this cascading influence may affect implementation of congruent culturally responsive practices. These findings extend knowledge regarding the role of leadership and climate in the implementation process in health care.

### Theoretical implications

Findings contribute to leadership theory on the embedded mechanisms that explain leadership influence on climate and practice implementation. Findings are consistent with emerging studies supporting the leadership–climate–practice mechanism in different organizational settings [14, 15, 78]. Because few studies have investigated the leadership process of implementation of cultural competence, our primary contribution lies in developing a measure of climate for implementation of cultural competence and identifying its relationship with the implementation of three validated measures of culturally responsive practices.

At the core of the leadership process related to influencing followers’ attitudes and behavior is the role of social exchange explained by social learning theory [79, 80]. However, additional embedded mechanisms play a role in influencing followers’ attitudes and behaviors. For instance, the social learning model relies on transformational leaders’ modeling behavior to emphasize principles (e.g., culture matters) and norms and behaviors (e.g., service providers should understand their clients’ culture to serve them effectively). These principles and norms may influence followers including middle managers, who in turn play a critical role in developing and maintaining an organizational climate focused on implementation of cultural competence [14, 15].

By considering additional embedded mechanisms and players in the social exchange dynamics described in social learning theory, this study highlights the importance of considering the hierarchical and role functions of transformational (executive) leaders and middle managers. Transformational leaders promote growth and a common vision [21, 81]. However, middle managers may need to translate this vision into specific priorities, norms, and behaviors that are actionable at the employee level. Executive leaders may value culture, whereas middle managers prioritize employee cross-cultural training and employees build their knowledge of clients’ cultural backgrounds and tailored service practices.

### Limitations and suggestions for future research

We acknowledge that our data have several limitations that should be considered when interpreting our findings. We did not test causal or temporal relationships because we relied on cross-section data, but informed by a conceptual framework. Second, program measures were provided by an average of three staff members per program, not including more individuals to indicate greater agreement on program climate for implementation of cultural competence can be a limitation. However, these outpatient clinics are generally small, independent, and similar to doctor’s offices. As such, these programs represent work environments that influence shared perceptions. We also acknowledge limitations on our measurement approach. We relied on individual respondent data and controlled for their nested structure instead of examining a true cross-level interaction relying only on measures separately aggregated at the counselor, supervisor, and director levels. Also, our measurement of climate for implementation, which relied on employees’ perceptions of supervisors’ priorities, or what is expected, rewarded, and promoted, needs to be further evaluated in terms of its discriminant validity. Emerging research has highlighted a strong relationship between middle managers’ reported commitment to innovation and implementation effectiveness [82, 83]. Distinguishing between middle-managers’ self-reported commitment to implementation and employees’ perceptions of their manager’s expectations, rewards, and priorities is critical to identifying different mechanisms of implementation.

Finally, we consider a limitation measuring implementation based on staff members’ reports of their program’s delivery or use of culturally responsive practices, rather than directly observing practices being implemented. Nonetheless, we relied on large, multilevel data from employees nested in programs and measures featuring different scales. This last issue reduces common methods bias and improves the rigor of our analysis.

## Conclusions

Findings underscore the empirical and theoretical importance of the leadership–climate relationship to implementing culturally competent practices in addiction health services. This is an important goal for these programs, which are located in one of the most ethnically diverse communities in the USA. Therefore, leadership development initiatives in health care can focus on teaching leaders to align incentives and communicate messages consistent with desired practices. Findings are relevant to executive leaders who may use a transformational leadership style (e.g., employee promotion) to influence midlevel supervisors’ implementation expectations and priorities. By doing so, program leaders can establish an effective leadership–climate–practice approach in their programs that strategically aligns directors’, supervisors’, and employees’ focus to enhance their organization’s capacity to implement culturally competent services for racial and ethnic minority clients.
